# Strain effects on rotational property in nanoscale rotation system

**DOI:** 10.1038/s41598-017-18903-9

**Published:** 2018-01-11

**Authors:** Jianzhang Huang, Qiang Han

**Affiliations:** School of Civil Engineering and Transportation, South China University of Technology, Guangzhou, 510640 China

## Abstract

This paper presents a study of strain effects on nanoscale rotation system consists of double-walls carbon nanotube and graphene. It is found that the strain effects can be a real-time controlling method for nano actuator system. The strain effects on rotational property as well as the effect mechanism is studied systematically through molecular dynamics simulations, and it obtains valuable conclusions for engineering application of rotational property management of nanoscale rotation system. It founds that the strain effects tune the rotational property by influencing the intertube supporting effect and friction effect of double-walls carbon nanotube, which are two critical factors of rotational performance. The mechanism of strain effects on rotational property is investigated in theoretical level based on analytical model established through lattice dynamics theory. This work suggests great potentials of strain effects for nanoscale real-time control, and provides new ideas for design and application of real-time controllable nanoscale rotation system.

## Introduction

Nanoelectromechanical system (NEMS) has pushed the revolution in modern industry of sensor, mechanical engineering and energy conversion system in last decade, because of the new physical properties and functions brought from its nanoscale feature sizes^1–4^. The research on NEMS has become one of the hottest subject throughout the world, thousands of research results of science and technology associated with NEMS have been published one after another. Carbon nanotube (CNT) and graphene, as excellent members among the carbon family with remarkable mechanical, chemical and thermoelectrical properties, have attracted great attention from the scientific community since their discovery. Their special geometric structure, robust in-plane mechanical property, relative low interlayer friction and good temperature stability make them promising ideal components in new class NEMS devices, such as nanoscale actuator, nano oscillator and nanogenerator. After Fennimore and Barreiro achieved to construct the nano actuator based on multi-walled carbon nanotube in laboratory^5,6^, growing efforts have been made for the design and fabrication of NEMS with carbon nanotube as main component. Among these published researches, there is no lack of innovative and fantastic ideas. For example, water molecules may be transported inside single carbon nanotube under temperature gradient^7^. Bailey *et al*. proposed a carbon nanotube based nanomotor called nano “windmills” driven by electrical flux^8^. Zhou *et al*. reported a numerical study of CNT charge-driven water pump^9^, and the pumping capacity could be controlled by the deformation of carbon nanotube. To speak frankly, restricted to current nanotechnology and nanofabrication technique, it is difficult to achieve these ideas in laboratory even for now. Although most researches on nano actuator are still limited to numerical simulations and theoretical studies, the published results may provide instructive and heuristic directions for design and application of the nano devices.

However, how to realize the real-time control of nano rotation is still an open issue. Nevertheless, to the best of our knowledge, the research on real-time control for the output performance of nano actuator has not been report so far. Real-time dynamical management of nano actuator is challenging problem in nanotechnology. Achieving real-time control only reduces the manufacturing cost and difficulty of operation, but also has great practical significance and engineering value on development of nanoscale actuator system.

At aspect of tuning the property of nano material and nano device, strain effect is likely to be most robust and simplest way to implement^10^. Actually, strain effects on material properties have been widely studied for a long time to exploiting superior properties of many functional materials and nanostructure, which is so-called strain engineering, such as magnetoelectricity, superconductivity and thermoelectricity^11–14^. Thermal conductivity of graphene ribbon may have remarkable decease after tensile or compressive strain is applied^15^. Tensile, compressive and torsional strains reduce the thermal conductivity of single-walled carbon nanotube respectively, and significant reduction of thermal conductivity occurs when the carbon nanotube buckles^16^. All researches mentioned above suggest the potential of strain effects for tuning the performance of nanodevice. This tuning mechanism, which has great impacts on the output of nano actuator, can be expediently implemented both in static and dynamic status. It is promising method to address the challenges of real-time control of nano actuator. Meanwhile, it is necessary to clearly understand the strain effects on the performance of nano actuator, as well as the effect mechanism. Because, in most cases, the nanodevices are subjected to stress/strains both in practical application and characterization in laboratory. In this paper, based on the nanoscale rotation systems proposed by Huang *et al*.^17^, molecular dynamics (MD) simulations are performed to systematically investigate the strain effects on of rotational property and effect mechanism. It shows that the real-time management of rotational property is successfully realized by strain effects. The relationship between tensile/compressive strain and rotational speed is obtained to draw engineering valuable conclusion. Analytical model is constructed to obtain further physical insight of strain effects on rotational property.

## Simulations model and methods

**Figure 1:**
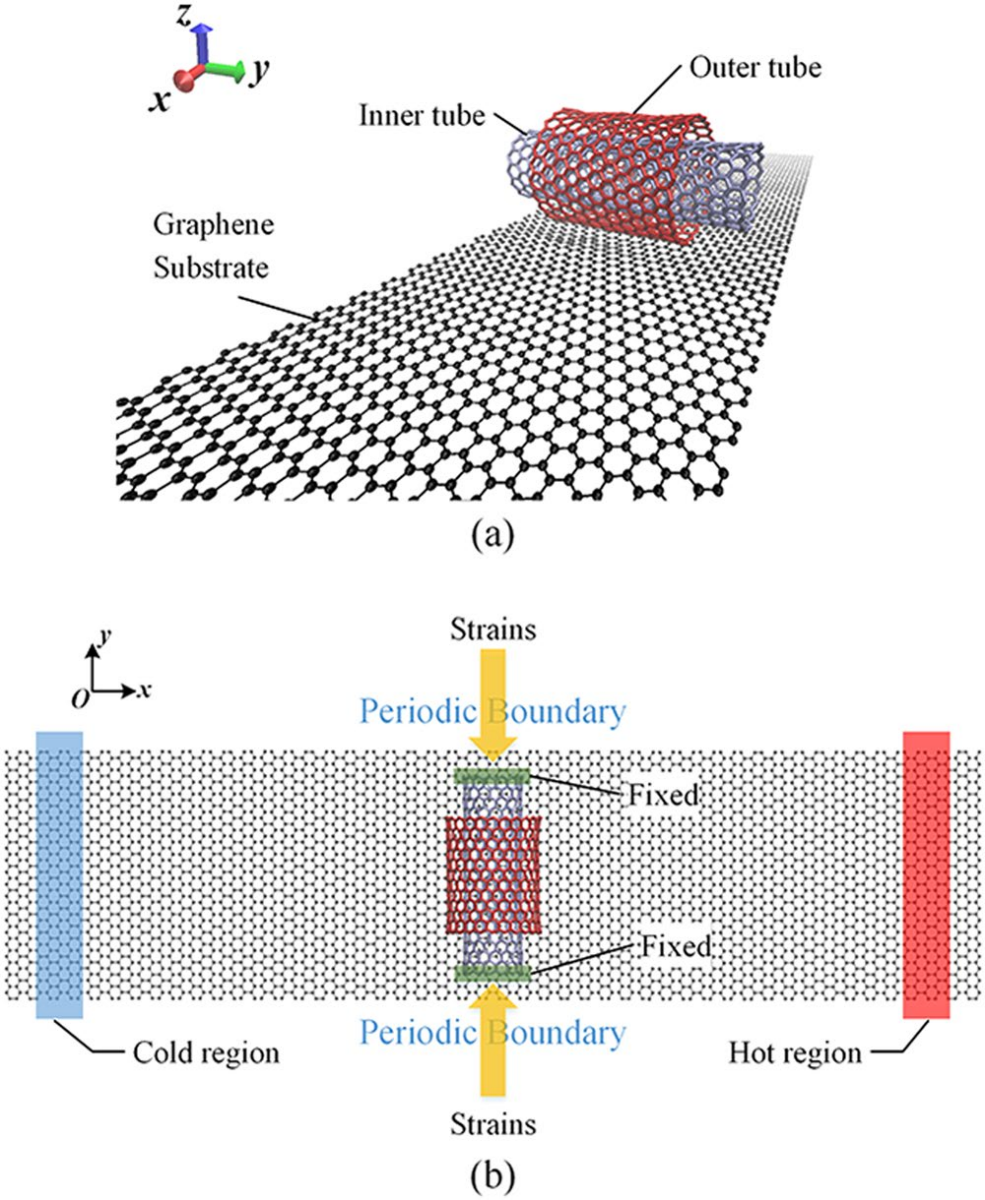
Schematic model of nanoscale rotation system consisting of graphene and DWCNT.

In the simulations, the double-walls carbon nanotube (DWCNT) with chirality registry of (15,0)/(24,0) and a single layer graphene with armchair edge along x direction are adopted to construct the rotation system, as shown in Fig. 1. The lengths of outer tube and inner tube are 2.5 and 4 nm respectively, and the size of single layer graphene is 20 × 5 *nm* ^2^. The distance between graphene and outer tube is 0.34 nm. This chirality assembly ensures easy rotation by lowering the energy barriers. The temperature gradient is applied to graphene as the driving power. Subjected to the temperature, the outer tube rotates around the inner tube which acts as supporting shaft.

**Figure 2:**
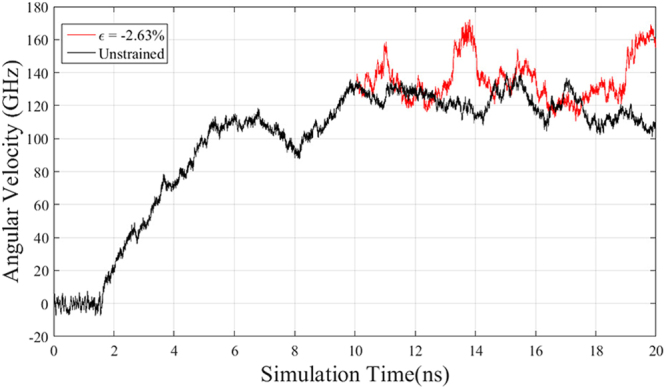
Rotation process of outer tube without strain effect shown as black line. Red line shows the rotation of outer tube with compressive strain of −2.63% applied at 10 ns.

According to the methodology of modeling pure rotation^17^, the outer tube exhibits pure rotation after the establishment of temperature gradient upon the graphene substrate. The angular velocity is stabilized around 117 GHz eventually as seen in Fig. 2. In the other case, an axial compressive strain of −2.63% along the y-direction is implemented to inner tube at 10 ns. It shows that the angular velocity (red line) is altered by the strain, indicating that strain effect can tune the rotational property dynamically, which can be regarded as a time-real control method. To save the computational cost, in all simulations, the strains are applied to inner tube before the establishment of temperature gradient, for intuitively investigating the strain effects on rotational property.

The MD simulations with time step of 1fs are performed using Large-scale Atomic/Molecular Massively Parallel Simulator (LAMMPS) package. The AIREBO potential^18^ is used to describe the interaction between carbon atoms. Periodic boundary condition is applied along the width direction of graphene. The atoms in two of ends of graphene and inner tube along the length direction are fixed through the whole simulation. Before simulation, the whole system containing 5208 atoms experiences a long-time relaxation under NVT ensemble at 300 K for energy minimization. The compressive and tensile strains with constant strain rate of 10^−2^ nm/ps are applied to inner tube by displacing the atoms in fixed region of both ends of the inner tube along the y-direction. A molecular dynamics relaxation is employed after every strain step. The strain is computed by *ε* = (*L* − *L*_0_)/*L*_0_, where the *L*_0_ and *L* is the initial and current length of the inner tube respectively. The values of compressive and tensile strains are listed in Tables 1 and 2, with negative for compressive strain and positive for tensile strain. After the strain is implemented, 200 ps relaxation is carried out at 300 K before building the temperature gradient along the graphene. Then the rotational signal of outer tube is obtained for investigation of the strain effects. The simulations are stopped running when the results are satisfied to meet the requirements.

**Table 1:** Angular velocity of rotation system under compressive strains.

Strain(%)	0	−2.11	−2.63	−3.42	−4.74	−5.26
Angular velocity(GHz)	117.22	138.35	169.51	103.67	86.66	81.05
Change (%)	0	18.02	44.60	−11.56	−26.07	−30.86

**Table 2:** Angular velocity of rotational system under tensile strains.

Strain(%)	0	1.32	3.95	5.26	7.89
Angular velocity(GHz)	117.22	126.15	154.54	95.73	73.99
Change (%)	0	7.62	31.83	−18.34	−36.88

## Results and Discussions

### Strain effects

**Figure 3:**
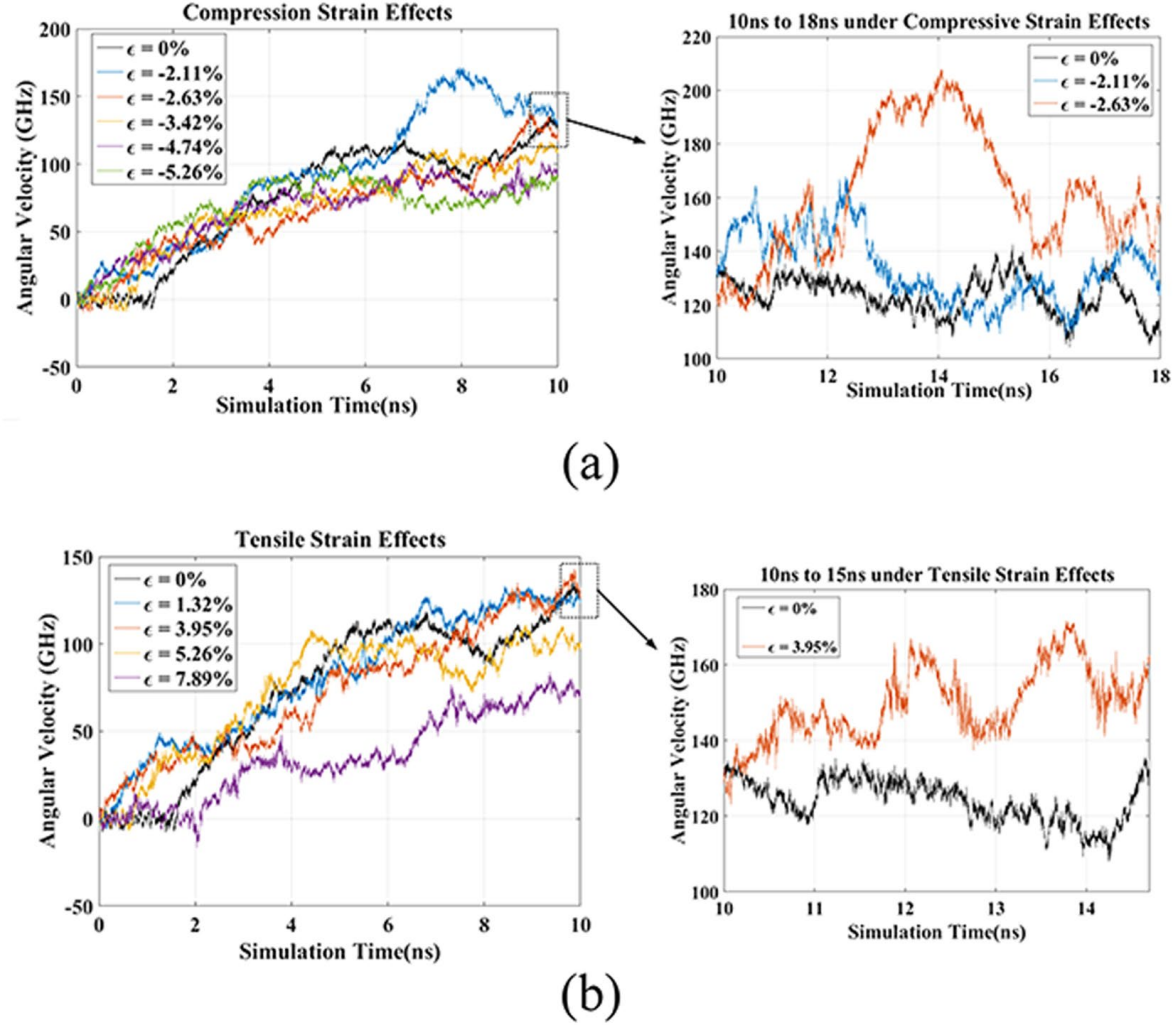
Angular velocity of rotation system under applied strains.

The angular velocity processes of the nano rotation system at strains are illustrated in Fig. 3. It shows that the strain effects have significant impact on the rotational performance. In such rotation system, the inner tube acts as a shaft to supports the outer tube, which proceeds fixed-axis rotation. The interaction between inner tube and outer tube, such as friction and support effect, are influenced by the inner tube strains, due to the atomic out-of-shell vibration of atoms in inner tube are affected by the applied strains. Strains change the relative position of atoms in carbon nanotube, that can alter the force constants based on lattice dynamic theory. As a result, the out-of-shell vibration of the atoms in carbon nanotube can be tuned by strains. And according to the analytical model built by Guo *et al*.^19^, the out-of-shell vibration of inner tube can affect the interaction between the inner tube and outer tube. According to the theoretical model^17^, the driving torque on outer tube has a direct correlation with the interaction effect between the outer tube and inner tube. It can be seen that the outer tube eventually rotates with steady angular velocity after it accelerated by the temperature gradient. In general, the whole process of rotation system from stationary to steadily rotation is divided into three periods, i.e. preparation period, acceleration period and the stabilization period. The preparation period can be found in previous work^17^, and it also occurred in the range of 1–2 ns. In the preparation period, the driving energy is accumulating to start the rotation of outer tube. It is observed that the strain effects affect the rotation throughout the whole process. The rotation process enters the acceleration period at around 1.5 ns in the case of without any strain. Under compressive strain, the rotation of outer tube enters to the acceleration period more quickly than unstrained case, and the latest entrance time for acceleration period of compressive strained cases is around 1.0 ns. It indicates that the compressive strain is beneficial to shorten the preparation period for practical application of rotation system. For tensile strain cases, the preparation period is reduced under low strains. However, it is elongated under relatively large tensile strain.

The average of angular velocity is calculated after acceleration stage as the stable angular velocity in each case for the convenience of discussion. The stable angular velocities at different strains are listed in Tables 1 and 2. It illustrates that the angular velocity rises initially then decreases as the strain increases, in both tensile and compressive strain cases. It is surprising that, it produces large influence on rotation of outer tube under such small strains. With compressive strain at −2.62%, the angular velocity reaches up to 169.51 GHz, producing 44.60% change in velocity, indicating it is really an efficient way to tune the rotational speed by strain effects. As the compressive increases to −3.42%, the rotational speed slows down and is lower than the one of unstrained case. The rotational speed reduces to 81.05 GHz at the strain of −5.26%, with fairly considerable adjustment rate of −30.86%. Compared to compressive strain, the adjustment efficiency of tensile strain on improving rotational speed is relatively lower, while at tensile strain of 3.95% the adjustment rate is 7.62.But as the tensile strain increases to 7.89%, the rotational speed is decreased to 73.99 GHz and the adjustment rate is −36.88%, demonstrating remarkable efficiency in reduction of rotational speed. The above findings suggest that it is more effective to apply compressive strain for improvement of rotational speed. On the contrary, implementing tensile strain is appropriate for slowing down the rotational speed. The tuning mechanisms found above have guiding significance to tuning nano rotation system using strain effects in practical application.

### Effect mechanism

**Figure 4:**
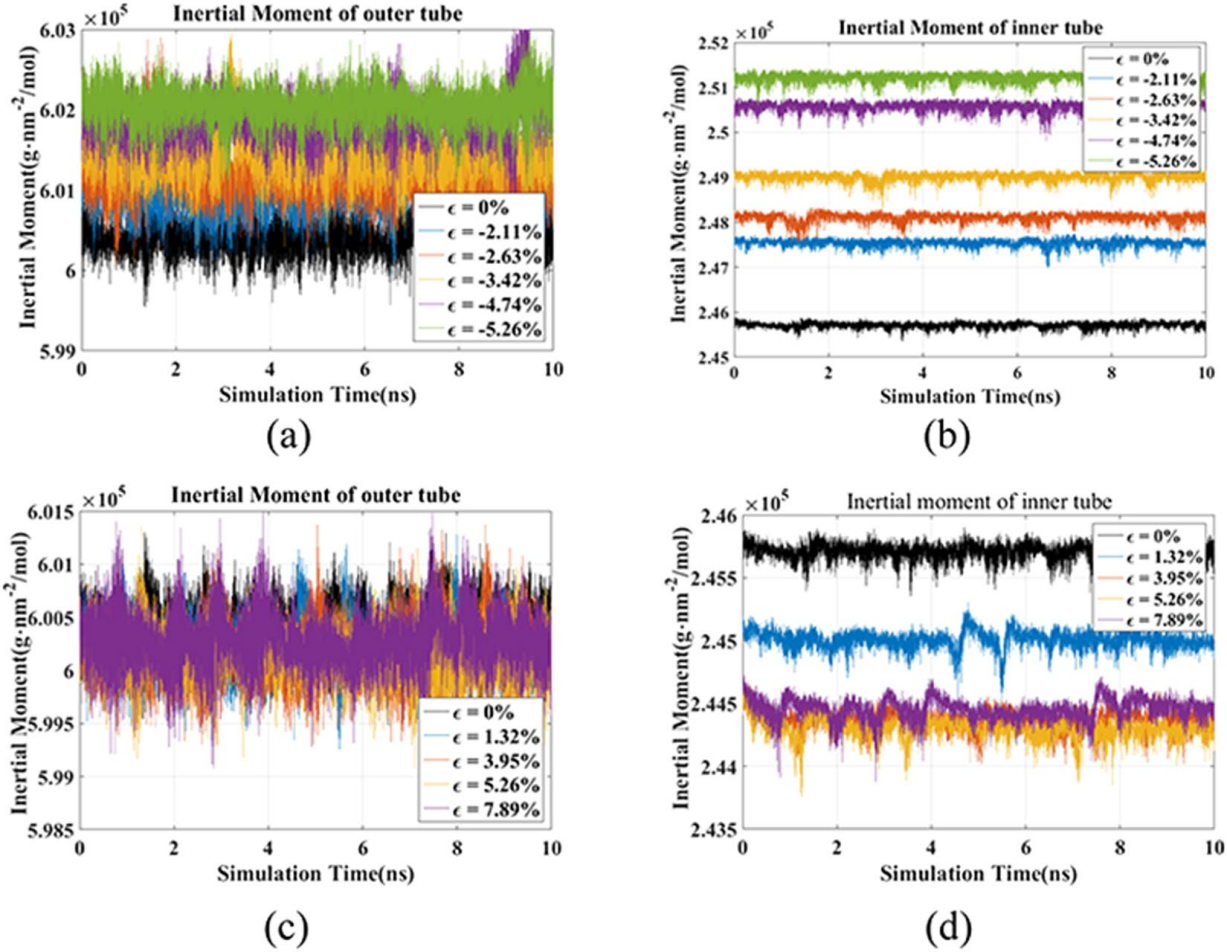
Inertial moment of the two tubes of DWCNT under different strains.

Revealing the mechanism of strain effect is extremely important for further understanding and method application of the strain effect tuning mechanism on rotation system. Figure 4 shows the moment of inertia of outer tube and inner tube along axis of rotation under compressive and tensile strains. The moment of inertia can be expressed by

1 $$J=\sum _{i=1}^{i=N}{m}_{i}{{r}_{i}}^{2}$$

where the *m*_*i*_ is the mass of carbon atoms and the *r*_*i*_ is the distance of atoms on CNT to the axis of rotation. The radius of the CNT during the dynamic process can be calculated through the equation above, and the result is shown in Tables 3 and 4.

**Table 3:** Radii of outer tube and inner tube under compressive strains.

Strain(%)	0	−2.11	−2.63	−3.42	−4.74	−5.26
Radius of outer tube (Å)	9.3203	9.3247	9.3262	9.3280	9.3318	9.3330
Radius of inner tube (Å)	5.8419	5.8636	5.8703	5.8810	5.8993	5.9069
Intertube distance (Å)	3.4784	3.4611	3.4559	3.4470	3.4325	3.4261

**Table 4:** Radii of outer tube and inner tube under tensile strains.

Strain(%)	0	1.32	3.95	5.26	7.89
Radius of outer tube (Å)	9.3203	9.3189	9.3184	9.3182	9.3192
Radius of inner tube (Å)	5.8419	5.8335	5.8260	5.8251	5.8269
Intertube distance (Å)	3.4784	3.4855	3.4924	3.4932	3.4923

The results illustrate that the radiuses of outer and inner tube are enlarged by the increasing compressive strain. Also, based on the nanomechanics theory, as the inner tube subjected to axial strains, according to the hyperelastic model for carbon nanotube developed by higher-order Cauchy-Born rule, the radius of the CNT increases with the increasing compressive strains^20^. Meanwhile, the intertube distance reduces as the radius of inner tube increases, resulting in the growth of van der Waals internal pressure to outer tube, which enlarges the radius of outer tube^21^.

The intertube distance reduces because the radius of inner tube grows faster than the outer tube’s. It shows that the intertube distance plays an important role in the effect mechanism. As the intertube distance decreases, the rotational speed first improves and then reduces. The supporting effect predominates at low strain cases, resulting in the improvement of the rotational speed. But the friction effect becomes prominent as the intertube distance decreases, therefore the rotational speed reduces.

When the tensile strain is applied, no substantial change observed in the radius (moment of inertia) of outer tube as the tensile strain increases. Obviously, the moment of inertia of the inner tube rapidly decreases when tensile strain starts loading. But after the tensile strain reaches a certain value, the moment of inertia of inner tube appears to be almost unchanged as the strain continues. Similar changing trend is shown in the intertube distance. The moment of inertia of inner tube should have reduced owing to the increasing tensile strain, but meanwhile the elongated C-C bonds in inner tube results in the aggravation of the out-of-shell atomic vibration, that give rise to the improvement of moment of inertia. So, the rotational speed decreases instead at higher strain with similar intertube distance, owing to the weakened supporting effect induced by aggravated atomic vibration. At low tensile strains, the rotational speed improves because the friction effect reduces resulting from the increased intertube distance. Due to poor supporting effect induced by high tensile strains, the rotational speed decreases as a result.

**Figure 5:**
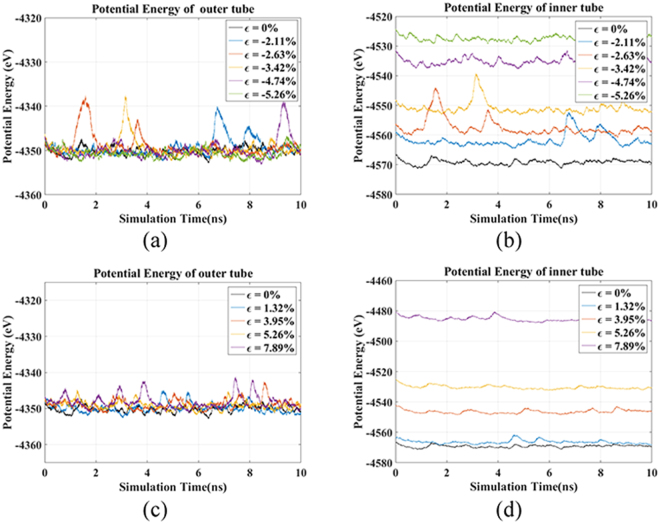
Potential energy of outer tube and inner tube under compressive and tensile strains.

The potential energies of two tubes in rotation system under different strains are shown in Fig. 5. It is observed that the energies are relatively stable during the whole simulation process except slight fluctuation at some times. The slight fluctuation caused by the thermal vibration of the graphene substrate returns to normal soon. The potential energy of inner tube increases because the strains are applied directly to the tube and leads to the increase of strain energy. Under the compressive strain, the radius of outer tube is enlarged by the squeezing of the increasing radius of inner tube, but the potential energy of outer tube remains unchanged with the increasing strains as shown in Fig. 5(a). It demonstrates that the outer tube keeps the potential energy at stable status by frictional consumption including changing the rotational speed^22^. On the other hand, the potential energy of outer tube stays stable as the radius does not vary with the tensile strains. It implies that the rotational speed is tuned by tensile strain through the supporting effect and friction effect.

### Theoretical analysis

From the results discussed above, it can be known that the applied strains tune the rotational speed by influencing the interaction between the outer tube and inner tube. Based on the analytical model^17^, the interaction between the two tubes can be described by the Van der Waals (VDW) interaction stiffness constant. Reasonable approximation is adopted to establish the expression of the interaction stiffness constant owing to the complication of the intertube VDW interaction. The intertube VDW interaction in DWCNT can be regarded as the interlayer VDW in double layered graphene for the diameter of the inner tube *D* ≥ 0.3 nm^23^. The Van der Waals interaction between two carbon atoms is described using Lennard-Jones (L-J) pair potential

2 $$V(r)=4\varepsilon (\frac{{\sigma }^{12}}{{r}^{12}}-\frac{{\sigma }^{6}}{{r}^{6}})$$

where *ε* = 0.0028 eV and *σ* = 0.341 nm. Using equivalent continuous approximation to replace the discrete structure, the VDW potential energy of a carbon caused by a monolayer graphene can be expressed by

3 $${\rm{\Phi }}=2\pi \rho \varepsilon {\sigma }^{2}(\frac{2{\sigma }^{10}}{5{s}^{10}}-\frac{{\sigma }^{4}}{{s}^{4}})$$

where $\rho =4\sqrt{3}\mathrm{/9}{{a}_{0}}^{2}$ is the carbon atom density, with the length of C-C bond of *a*_0_ and the distance of carbon to monolayer graphene of *s*.

According to the lattice dynamics theory, the VDW interaction stiffness constant can be deduced as

4 $${k}_{vdw}({a}_{0},s)=\frac{{\partial }^{2}{\rm{\Phi }}({a}_{0},s)}{\partial {s}^{2}}=\frac{8\sqrt{3}\pi \varepsilon {\sigma }^{2}}{9{{a}_{0}}^{2}}(\frac{44{\sigma }^{10}}{{s}^{12}}-\frac{20{\sigma }^{4}}{{s}^{6}})$$

It shows that the stiffness constant is the function of bond length *a*_0_ and relative distance *s*.

**Figure 6:**
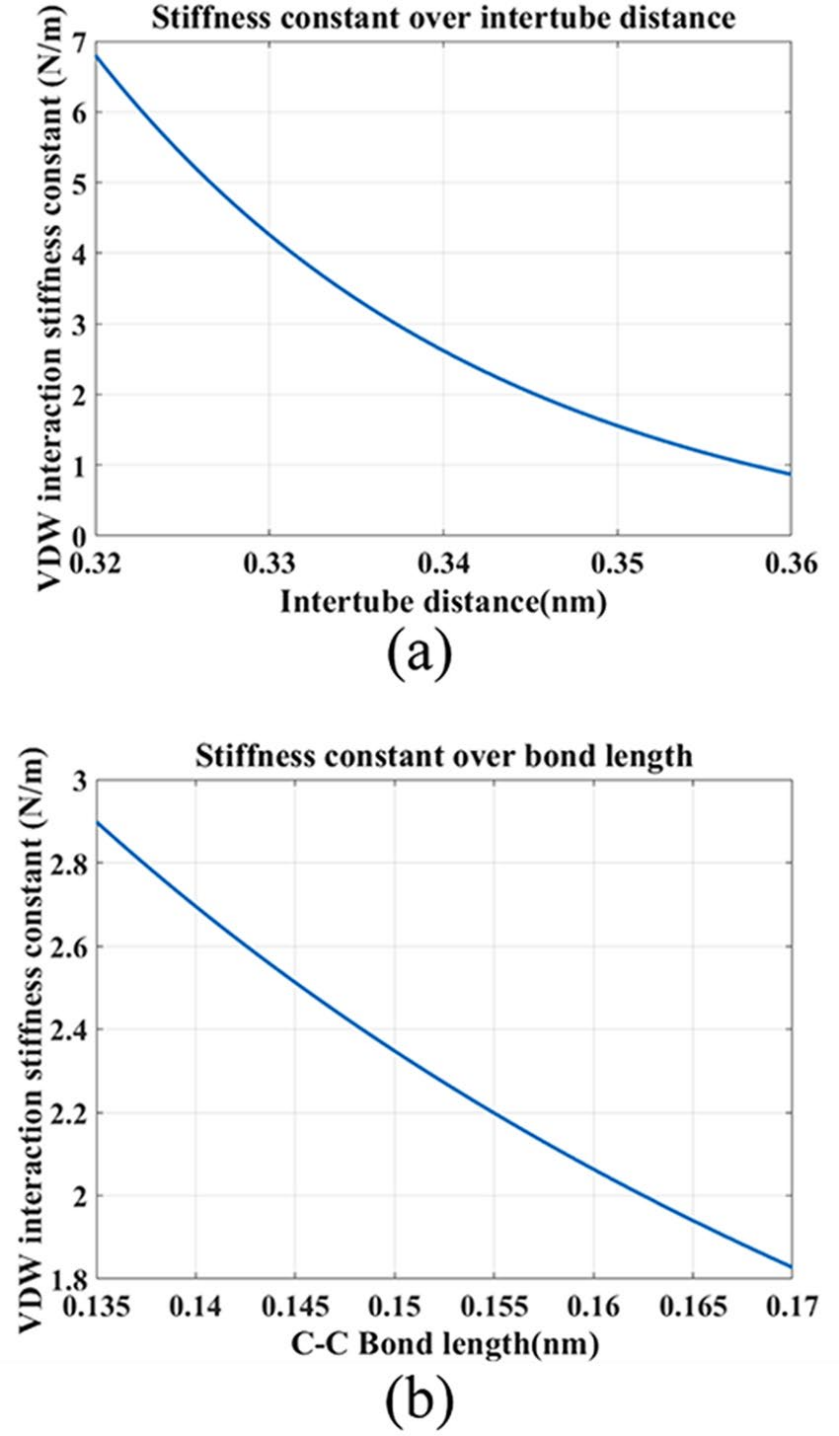
Stiffness constant over intertube distance and C-C bond length. (**a**) with *a*_0_ = 0.142 nm, (**b**) with *s* = 0.34 nm.

The numerical results of stiffness constant *k*_*vdw*_ over bond length *a*_0_ and relative distance *s* are illustrated in Fig. 6. It can be seen that the stiffness constant is more sensitive to relative distance rather than the C-C bond length, indicating relatively great impact of intertube distance on the stiffness constant. With *a*_0_ = 0.142 nm, when *s* = 0.34 nm, the *k*_*vdw*_ = 2.62 N/m, while the *s* equals to 0.33 nm and 0.35 nm, the *k*_*vdw*_ becomes 4.26 N/m and 1.56 N/m, implying large difference. Comparatively speaking, the stiffness constant is less affected by bond length as shown in Fig. 6(b).

When the compressive strains are implemented, the C-C bonds in nanotube are compressed and the intertube distance is reduced, that enhance the intertube interaction. Meanwhile the radius of outer tube increases and well contacts with the temperature-gradient graphene, making the rotational speed improves. As the compressive strain grows, the friction effect increases remarkably due to the strengthened intertube interaction, resulting in the drop of the rotational speed. In the tensile strain cases, the C-C bonds length and the intertube distance are extended, the influence (e.g. friction) of the inner tube on outer tube are weaken, so there is an increase of rotational speed. As the of tensile strain raises, the supporting effect of inner tube to outer tube recedes with the increase of intertube distance, that lower the rotational stability. Therefore, the rotational speed slows down at high tensile strain. The strain effects on rotational performance of rotation system are analyzed in theory to obtain physical insight in the section.

## Conclusion

In this paper, the strain effects on the rotational property of nanoscale rotation system, which can be utilized as the real-time control method in nanoscale, are systematically investigated by molecular dynamics simulations and analytical model built on lattice dynamics theory. It is found that strain effects highly effectively tune the rotational speed of the system in a wide range, and the rotational speed improves under low strains then reduces under high strains. Furthermore, the compressive strains are beneficial for improvement of the rotational speed while the tensile strains are preferable for slowing down the speed, which is valuable conclusion for engineering application. The investigation of tuning mechanisms shows that the strain effects adjust the rotation by affecting the supporting effect and friction effect, which are the key factors in nanoscale rotation system. Through analytical studies, it indicates that strains tune the rotational speed through the intertube interaction by altering the C-C bond length and intertube distance. The analytical model shows that the intertube interaction is more sensitive to intertube distance than C-C bond length. The present work has important implication of the dynamic performance management of the nano actuator utilizing the strains effects.
